# Future Confidence and Self-Reported Health Among the Bereaved Elderly in China

**DOI:** 10.1093/geroni/igaa057.544

**Published:** 2020-12-16

**Authors:** Haimin Pan, Qian Liu

**Affiliations:** 1 Xiamen University, Xiamen, China; 2 Hunan Normal University, Changsha, China

## Abstract

Time perspective serves as an important determinant for the elderly’s health-related outcomes, including those in bereavement. Confidence in the future (CF) seems a reflecting viewpoint a in this line, however, it is hardly investigated as a predictor for health among the elderly. This study centered on the casual relationship between CF and self-reported health (SRH) among the bereaved older Chinese adults. Data from a sample of 376 elders in bereavement were finally used for analysis. The sample was extracted from a national representative data, namely, China Family Panel Studies (CFPS), and contained 2012, 2014, 2016, and 2018 waves of information. A across-lagged model with four time points was used to explore the longitudinal relationship between CF and SRH among the bereaved elderly in China. This model achieved acceptable indices of goodness of fit, and the results revealed that SRH positively predicted future confidence in general among the senior participants, while CF failed to predict SRH at any point of time. The cross-sectional associations between CF and SRH were generally positively significant. The results implied the casual effect of SRH on CF, which was different from the cross-sectional relationship between CF and SRH. It seemed that enhancing the oral health of the bereaved elderly could be beneficial for increase in their CF. Tailored services are recommended for improving the perception of the bereaved elderly on their general health, and thus strengthening their positive perspectives for future.

